# [^64^Cu]XYIMSR-06: A dual-motif CAIX ligand for PET imaging of clear cell renal cell carcinoma

**DOI:** 10.18632/oncotarget.10602

**Published:** 2016-07-14

**Authors:** Il Minn, Soo Min Koo, Hye Soo Lee, Mary Brummet, Steven P. Rowe, Michael A. Gorin, Polina Sysa-Shah, William D. Lewis, Hye-Hyun Ahn, Yuchuan Wang, Sangeeta Ray Banerjee, Ronnie C. Mease, Sridhar Nimmagadda, Mohamad E. Allaf, Martin G. Pomper, Xing Yang

**Affiliations:** ^1^ Russell H. Morgan Department of Radiology and Radiological Science, Johns Hopkins University School of Medicine, Baltimore, MD, USA; ^2^ The James Buchanan Brady Urological Institute and Department of Urology, Johns Hopkins University School of Medicine, Baltimore, MD, USA

**Keywords:** carbonic anhydrase IX, positron emission tomography, molecular imaging, renal cell carcinoma, copper-64

## Abstract

Carbonic anhydrase IX (CAIX) is a cell surface enzyme that is over-expressed in approximately 95% of cases of clear cell renal cell carcinoma (ccRCC), the most common renal cancer. We synthesized and performed *in vitro* and *in vivo* evaluation of a dual-motif ligand, [^64^Cu]XYIMSR-06, for imaging CAIX expression on ccRCC tumors using positron emission tomography (PET). [^64^Cu]XYIMSR-06 was generated in yields of 51.0 ± 4.5% (n=5) and specific activities of 4.1 – 8.9 GBq/μmol (110-240 Ci/mmol). Tumor was visualized on PET images by 1 h post-injection with high tumor-to-background levels (>100 tumor-to-blood and -muscle) achieved within 24 h. Biodistribution studies demonstrated a maximum tumor uptake of 19.3% injected dose per gram of radioactivity at 4 h. Tumor-to-blood, -muscle and -kidney ratios were 129.6 ± 18.8, 84.3 ± 21.0 and 2.1 ± 0.3, respectively, at 8 h post-injection. At 24 h a tumor-to-kidney ratio of 7.1 ± 2.5 was achieved. These results indicate pharmacokinetics superior to those of previously reported imaging agents binding to CAIX. [^64^Cu]XYIMSR-06 is a new low-molecular-weight PET ligand targeting CAIX, which can image localized and metastatic ccRCC.

## INTRODUCTION

Clear cell renal cell carcinoma (ccRCC) is the most common neoplasm of the kidney, accounting for up to 70% of renal cell carcinomas (RCCs) [1–3]. When small (< 4 cm) and localized to the kidney, ccRCCs are indistinguishable from other renal tumor histologies (including benign tumors and other indolent subtypes of RCC) on the basis of either anatomic imaging or [^18^F]fluorodeoxyglucose (FDG) positron emission tomography (PET) [4–7]. Although renal mass biopsy can aid in distinguishing between the various renal tumor histologies, this procedure is often forgone due to a relatively high rate of non-diagnostic biopsies and the potential for complications [8]. Thus, the majority of patients presenting with a small renal mass undergo surgical resection for the presumption of cancer without first undergoing a biopsy [9].

Surgery with either partial or radical nephrectomy is currently the mainstay of treatment for ccRCC localized to the kidney. Although the majority of patients presenting with non-metastatic ccRCC are cured with surgery alone, up to 30% will experience a recurrence. Such recurrence can manifest late (i.e. >10 years) after resection and tumors may arise in unusual sites that can confound detection [10, 11]. Given these considerations, there exists a clinical need for improved imaging of ccRCC. Potential applications include the ability to distinguish ccRCC from other renal tumor histologies, guidance of extent of surgical resection, and reliable detection of locally recurrent and/or metastatic disease [12, 13].

Carbonic anhydrase IX (CAIX) is an attractive target for the diagnosis and targeted therapy of ccRCC. CAIX is a membrane-associated member of the carbonic anhydrase (CA) family, with normal tissue expression restricted to the gastrointestinal tract, gallbladder and pancreatic ducts [14, 15]. Over-expression of CAIX has been observed in approximately 95% of ccRCC tumor specimens due to common loss of the von Hippel-Lindau (VHL) tumor suppressor gene [16–18]. As one of the downstream proteins regulated by hypoxia-inducible factor-1α (HIF-1α), CAIX plays an important role in homeostasis of tumor pH [19, 20]. In fact, levels of CAIX expression have been reported as an independent predictor of survival in advanced ccRCC [16].

To date, a number of radiotracers have been developed for CAIX imaging. Most notably, the ^124^I-labeled chimeric antibody cG250 has demonstrated excellent sensitivity and specificity for PET imaging of ccRCC in clinical studies [21–23]. However, there exist significant limitations for the widespread application of this imaging agent. These include the long circulating time required for tumor resolution, high associated cost associated with antibody production, and issues with deiodination [24]. As an alternative, a low molecular weight (LMW) imaging agent targeting CAIX would likely have greater clinical applicability. Such agents would have faster pharmacokinetics and could allow for imaging within a more convenient timeframe after radiotracer administration [25, 26]. LMW agents also have advantages in synthesis, purification, and a shorter path to regulatory approval [27].

Recently we reported the synthesis of a dual-motif LMW single-photon emission computed tomography (SPECT) radioligand, [^111^In]XYIMSR-01 [28], targeting CAIX. This radiotracer was initially discovered from a DNA-encoded chemical library [30–32] and demonstrated excellent *in vitro* selectivity for CAIX [28]. In addition, [^111^In]XYIMSR-01 significantly improved both target-selective *in vivo* radiotracer uptake and pharmacokinetic properties compared to other reported ligands [29, 33–37]. More specifically, [^111^In]XYIMSR-01 demonstrated 26% injected dose per gram (ID/g) of uptake within tumor at 1 h post-injection, and tumor-to-blood, -muscle and -kidney ratios of 178.1 ± 145.4, 68.4 ± 29.0 and 1.7 ± 1.2 at 24 h, with the potential to image both metastatic and localized ccRCC [28]. Given the promising imaging parameters of [^111^In]XYIMSR-01, and in order to leverage the inherently higher sensitivity and resolution of PET [38], we synthesized and evaluated a positron-emitting, dual-motif radioligand targeting CAIX.

## RESULTS AND DISCUSSION

**Figure 1:**
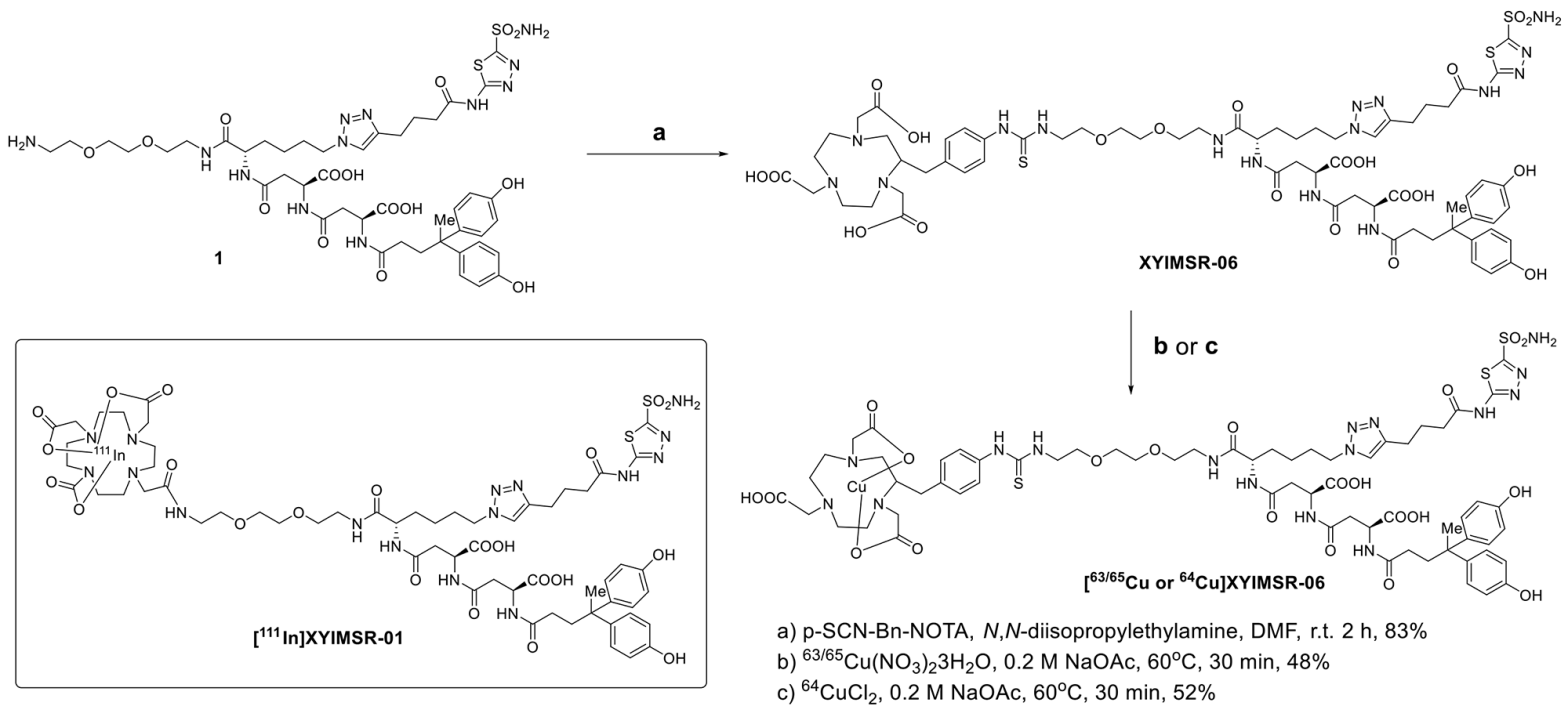
Structure of [^111^In]XYIMSR-01 and the synthesis of [^63/65^Cu/^64^Cu]XYIMSR-06

**Figure 2:**
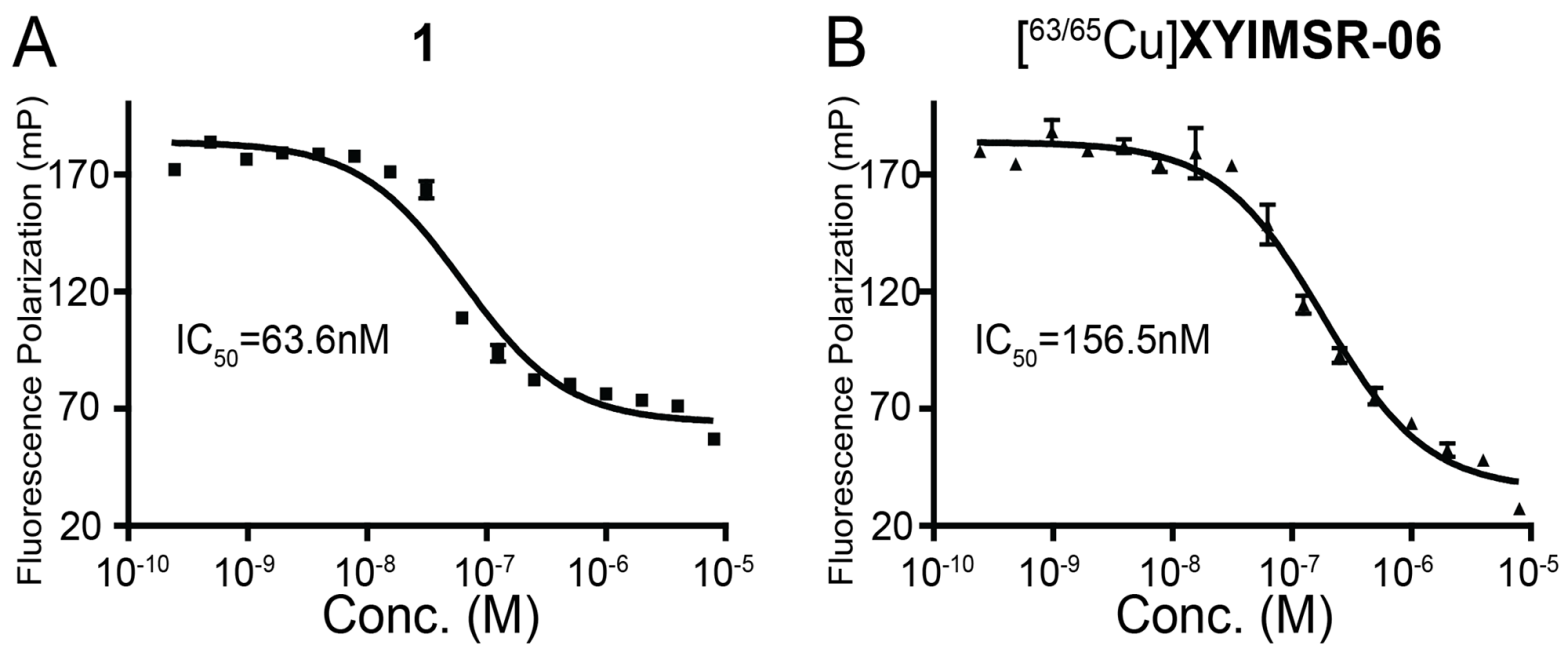
[^63/65^Cu]XYIMSR-06 demonstrates high binding affinity to CAIX IC_50_ values of positive control 1 and [^63/65^Cu]XYIMSR-06 were determined by measuring inhibition of fluorescence polarization of a corresponding FITC-labeled dual-motif ligand [28].

The recent discovery of the 4,4-bis(4-hydroxyphenyl)valeric acid/acetazolamide dual-motif CAIX ligand [30] has enhanced the development of targeted SPECT imaging of ccRCC in a preclinical model [28]. The hydrophilic DOTA-In(III) chelate not only provided a platform for convenient labeling with radio-metals for imaging and therapeutic applications [39, 40], but also provided salutary pharmacokinetic properties for the radiotracer. [^111^In]XYIMSR-01 achieved high tumor uptake and high tumor-to-background ratios. Given the superiority of PET over SPECT with respect to sensitivity, resolution, and quantification for clinical imaging, we initially investigated ^18^F as the radionuclide to incorporate into the CAIX targeting scaffold. We synthesized [Al^18^F]XYIMSR-04, with details noted in the Supplemental Data. The binding of [Al^19^F]XYIMSR-04 to CAIX was evaluated using a competitive fluorescence polarization assay with a previously described FITC-labeled dual-motif ligand [30] and 1 (Supplementary Figure S1). The IC_50_ values determined for 1 and [Al^19^F]XYIMSR-04 were 63.6 ± 2.8 and 96.7 ± 3.3 nM, respectively (Figure 2 and Supplementary Figure S1). Compound 1 was used as positive control, which has been reported with an IC_50_ value of 75.9 nM using the same method [28]. PET imaging (Supplementary Figure S2) and biodistribution studies (Supplementary Table S1) at 1 h post-injection of [Al^18^F]XYIMSR-04 to mice bearing CAIX-expressing SK-RC-52 ccRCC tumor xenografts showed 14.4 ± 2.2 %ID/g tumor uptake. Tumor-to-blood, -muscle and -kidney ratios were 21.1 ± 1.5, 9.7 ± 2.9 and 0.28 ± 0.03, respectively. Although the 110 min half-life of ^18^F is ideal for many PET radiotracers, it would not allow for sufficient time for the radioligand to clear from the kidneys to permit adequate visualization of ccRCC localized to the kidney. We therefore switched our focus to ^64^Cu, which has a half-life of 12.7 h, enabling such clearance. We chose 2-S-(4-isothiocyanatobenzyl)-1,4,7-triazacyclononane-1,4,7-triacetic acid (p-SCN-Bn-NOTA), XYIMSR-06, as the scaffold to house ^64^Cu (Figure 1). We hypothesized that the NOTA chelator would provide stable copper chelation *in vivo*, as we observed earlier in other studies [41] and the one extra free carboxylate would further increase the hydrophilicity for faster clearance from normal tissues.

Chemical synthesis of [^63/65^Cu]XYIMSR-06 is shown in Figure 1. Commercially available p-SCN-Bn-NOTA was conjugated with the dual-motif CAIX binding precursor 1 to afford XYIMSR-06 in 83 % yield. [^63/65^Cu]XYIMSR-06 was obtained in 48 % yield by heating XYIMSR-06 with copper(II) nitrate at 60°C in 0.2 M NaOAc buffer. We determined the binding affinity of [^63/65^Cu]XYIMSR-06 to CAIX as described above. The IC_50_ values determined for 1 and [^63/65^Cu]XYIMSR-06 were 63.6 ± 2.8 and 156.5 ± 4.3 nM, respectively (Figure 2). Radiosynthesis of [^64^Cu]XYIMSR-06 was undertaken by adding 40 μg of XYIMSR-06 to 155.4-255.3 MBq (4.2-6.9 mCi) ^64^CuCl_2_ in a total volume of 80 μL NaOAc at 65°C for 0.5 h. After purification by high performance liquid chromatography (HPLC), [^64^Cu]XYIMSR-06 was obtained in yields of 51.0 ± 4.5 % (non-decay corrected) with specific activities of 4.1 – 8.9 GBq/μmol (110-240 Ci/mmol) (n=5). Total synthesis time was less than 1.5 h. The high yield and specific activity were suitable for preclinical studies.

**Figure 3:**
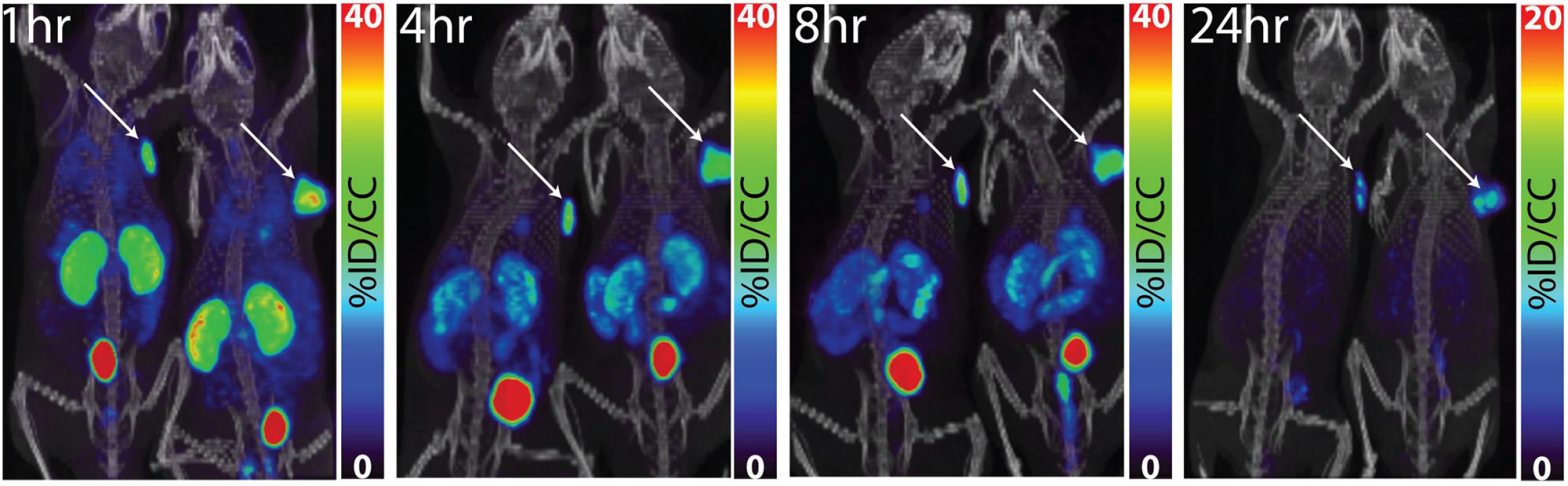
Whole-body PET/CT images of [^64^Cu]XYIMSR-06 enabled the detection of CAIX-expressing tumors *in vivo* Representative whole PET/CT images of two mice bearing SK-RC-52 tumors within the upper right flank. Images were obtained at 1, 4, 8, and 24 h after injection of 22.2 MBq (600 μCi) of [^64^Cu]XYIMSR-06 *via* the tail vein. Arrows indicate tumors. Scales were adjusted to percent injected dose per volume (%ID/cc).

[^64^Cu]XYIMSR-06 was assessed in immunocompromised mice bearing CAIX-expressing SK-RC-52 ccRCC tumor xenografts as described previously [42]. PET was performed at 1, 4, 8 and 24 h post-injection of 22.2 MBq (600 μCi) of [^64^Cu]XYIMSR-06 in 2 mice bearing SK-RC-52 ccRCC tumor xenografts, as shown in Figure 3. At 1 h tumor could be observed distinctly with additional visible signal in the kidneys and urinary bladder. Relatively selective tumor imaging could be achieved at 8 h with target selectivity continuing to improve by 24 h, with the SK-RC-52 ccRCC tumor xenografts as the only remaining visible radiotracer-avid structures. There was no significant background signal from blood or muscle. The liver did not retain significant radioactivity at any time.

Following imaging, biodistribution was formally quantified with 5 animals per time point as previously described [28]. Table 1, Supplementary Table S2 and Supplementary Figure S3 show the radiotracer uptake and biological half-life (Supplementary Table S2) in selected organs. Radiotracer uptake within the tumor was 14.5 %ID/g at 1 h with tumor-to-blood and -muscle ratios >10. After the radiotracer reached a maximum of 19.3 %ID/g in tumor at 4 h, it began to wash out from tumors slowly. By 24 h radioactivity within the tumors dropped to 6.2 %ID/g. Compared with [^111^In]XYIMSR-01 (20.8 %ID/g at 4 h, 34.0 %ID/g at 8 h, 25.6 %ID/g at 24 h and 13.9%ID/g at 48 h), [^64^Cu]XYIMSR-06 demonstrated faster clearance, likely due to the more hydrophilic nature of NOTA-Cu(II), which has an additional non-coordinated free carboxylate not present for DOTA-In(III). At 8 h post-injection tumor signal was predominant, with kidney, lung and stomach as the only readily visible organs. Tumor-to-blood, -muscle and -kidney ratios were 129.6 ± 18.8, 84.3 ± 21.0 and 2.1 ± 0.3, respectively. In principle those ratios would allow the detection of localized tumor in kidney. At 24 h, tumor-to-kidney and -lung ratios were further improved to 7.1 and 4.9, respectively, with all other tumor-to-organ ratios tested ≥ 10. Co-injection of 200 nmole of 1 along with [^64^Cu]XYIMSR-06 blocked tumor uptake of the latter (Table 1) indicating specific, CAIX-mediated binding of this radiotracer. Within 24 h, no significant radiotracer uptake within liver was observed, indicative of the *in vivo* stability of NOTA-^64^Cu chelation.

**Table 1:** Biodistribution of [^64^Cu]XYIMSR-06

Organs	1 h	4 h	8 h	8 h+Block	24 h	24 h+Block
Blood	0.66±0.05	0.33±0.06	0.13±0.02	0.08±0.01	0.00±0.06	0.01±0.08
Heart	3.41±0.79	1.78±0.33	0.65±0.10	0.16±0.03	0.12±0.03	0.08±0.01
Lung	25.52±3.35	12.03±1.61	4.64±0.58	0.77±0.14	1.27±0.38	0.29±0.05
Pancreas	2.94±0.24	1.36±0.19	0.47±0.21	0.14±0.03	0.21±0.29	0.07±0.02
Spleen	0.36±0.08	0.24±0.04	0.18±0.02	0.14±0.02	0.09±0.01	0.11±0.04
Fat	0.80±0.17	0.52±0.29	0.28±0.27	0.05±0.01	0.03±0.01	0.01±0.01
Brain	1.46±2.23	0.29±0.06	0.24±0.06	0.04±0.01	0.13±0.02	0.02±0.01
Muscle (mm)	1.28±0.27	0.70±0.18	0.21±0.05	0.05±0.02	0.02±0.01	0.04±0.06
Sm. intestine	7.35±0.78	4.15±0.69	1.98±0.14	0.38±0.06	0.47±0.03	0.19±0.05
Liver	3.25±0.97	2.18±0.57	0.99±0.22	0.55±0.08	0.38±0.03	0.42±0.04
Stomach	14.80±2.27	8.19±0.97	4.02±0.53	0.69±0.18	0.65±0.04	0.29±0.04
Kidney (kid)	33.65±3.91	19.99±2.88	8.14±1.15	1.82±0.39	0.90±0.12	0.48±0.09
Bladder	11.12±6.33	17.05±8.86	7.43±7.72	1.45±1.53	0.34±0.09	0.45±0.50
Tumor	14.47±2.69	19.31±4.51	16.74±1.58	2.39±0.24	6.23±1.41	1.20±0.47
Tumor/Blood	21.9±4.6	57.7±9.3	129.6±18.8	32.0±3.8	142.6±115.8	27.1±26.7
Tumor/mm	10.8±3.7	29.4±9.9	84.3±21.0	53.0±12.6	261.3±47.3	49.0±28.7
Tumor/kid	0.4±0.1	1.0±0.1	2.1±0.26	1.3±10.2	7.1±2.5	2.4±0.4

Results are expressed as the percentage injected dose per gram (%ID/g) of tissue, *n* = 5 per time point.

Compared with [^111^In]XYIMSR-01 the faster clearance of [^64^Cu]XYIMSR-06 could be a result of its increased hydrophilicity, especially with the lack of internalization of CAIX upon ligand binding [28, 43]. This increase in radiotracer clearance led to improved tumor conspicuity relative to normal tissues including the kidney. As shown in Table 1, the tumor-to-kidney ratio was nearly 1.0 at 4 h, and increased to 2.1 at 8 h, and 7.1 at 24 h. This is in sharp contrast to [^111^In]XYIMSR-01, which had a ratio of 1.7 at 24 h. These data suggest the potential utility of [^64^Cu]XYIMSR-06 in imaging ccRCC localized to the kidney. Other significant normal organ uptake of [^64^Cu]XYIMSR-06 was observed in bladder, lung and stomach, similar to [^111^In]XYIMSR-01, although with overall relatively faster wash-out in comparison to the ^111^In-labeled radiotracer. With respect to the potential assessment of metastatic lesions, the faster off-target wash-out would complement the otherwise intrinsically enhanced sensitivity and spatial resolution of a PET radiotracer. Blocking studies with compound 1 at 8 h and 24 h, proved CAIX-mediated binding of [^64^Cu]XYIMSR-06. [^64^Cu]XYIMSR-06 was found to be stable up to 2 h post-injection analyzed by blood metabolites using radio thin layer chromatography (Supplementary Figure S4).

Relative to the radiolabeled antibody, ^124^I-cG250 [21, 44], which has been studied extensively, the LMW ligand [^111^In]XYIMSR-01 demonstrated advantages with respect to blood and normal tissue clearance [28]. [^64^Cu]XYIMSR-06 represents a further improvement on [^111^In]XYIMSR-01 in both imaging mechanism (PET vs. SPECT) and pharmacokinetic profile. Additional preclinical work with compounds of this class will seek to refine pharmacokinetics and image quality.

## CONCLUSION

We describe a new LMW, dual-motif, positron-emitting, CAIX-targeted radiotracer, [^64^Cu]XYIMSR-06, with potential applications for imaging localized and metastatic ccRCC. [^64^Cu]XYIMSR-06 demonstrated selective tumor uptake and pharmacokinetic properties suitable for clinical imaging. [^64^Cu]XYIMSR-06 provides a clinically viable alternative for molecular imaging of ccRCC.

## EXPERIMENTAL SECTION

### General chemistry and radiochemistry methods

Solvents and chemicals obtained from commercial sources were of analytical grade or better and were used without further purification. Fmoc-protected azidolysine, HBTU, and N-α-fmoc-L-aspartic acid α-tert-butyl ester were purchased from Chem Impex International, Inc. (Wooddale, IL). Na^18^F in saline was purchased from PETNET (Hackensack, NJ). [^64^Cu]CuCl_2_ was purchased from the University of Wisconsin. p-SCN-Bn-NOTA, was purchased from Macrocyclics, Inc. (Dallas, TX). NOTA-NHS ester was purchased from CheMatech(Dijon, France). Copper (II) nitrate, triethylsilane (Et_3_SiH), *N*, *N*-diisopropylethylamine (DIEA), triethylamine (TEA), piperidine, 4,4-bis(4-hydroxyphenyl)valeric acid, copper iodide (CuI), and tris[(1-benzyl-1H-1,2,3-triazol-4-yl)methyl]amine (TBTA) were purchased from Sigma-Aldrich (Saint Louis, MO). Pre-loaded O-bis-(aminoethyl)ethylene glycol on trityl resin was purchased from EMD Millipore (Billerica, MA). Flash chromatography was performed using MP SiliTech 32-63 D 60Å silica gel purchased from Bodman (Aston, PA). Recombinant human CAIX was purchased from R&D Systems (Minneapolis, MN). ^1^H-NMR spectra were recorded on a Bruker Ultrashield 500 MHz spectrometer. Chemical shifts (δ) were reported in ppm downfield by reference to proton resonances resulting from incomplete deuteration of the NMR solvent. ESI mass spectra were obtained on a Bruker Daltonics Esquire 3000 Plus spectrometer (Billerica, MA).

HPLC purification of non-labeled compounds was performed using a Phenomenex C18 Luna 10 × 250 mm^2^ column on an Agilent 1260 infinity LC system (Santa Clara, CA). HPLC purification of radiolabeled ligands was performed on another Phenomenex C18 Luna 10 × 250 mm^2^ and a Varian Prostar System (Palo Alto, CA), equipped with a Varian ProStar 325 UV-Vis variable wavelength detector and a Bioscan (Poway, CA) Flow-count in-line radioactivity detector, all controlled by Galaxie software. The specific radioactivity was calculated as the ratio of the radioactivity eluting at the retention time of product during the preparative HPLC purification to the mass corresponding to the area under the curve of the UV absorption. The purity of tested compounds as determined by analytical HPLC with absorbance at 254 nm was > 95%.

### Synthesis and characterization

2,2′,2″-(2-(4-(3-((11S,15S,19S)-15,19-Dicarboxy-24,24-bis(4-hydroxyphenyl)-10,13,17,21-tetraoxo-11-(4-(4-(4-oxo-4-((5-sulfamoyl-1,3,4-thiadiazol-2-yl)amino)butyl)-1H-1,2,3-triazol-1-yl)butyl)-3,6-dioxa-9,12,16,20-tetraazapentacosyl)thioureido)benzyl)-1,4,7-triazonane-1,4,7-triyl)triacetic acid (XYIMSR-06). 1 [30] 12 mg (0.0111 mmol), p-SCN-Bn-NOTA 8 mg (0.0143 mmol) and *N*,*N*-diisopropylethylamine 50 μL were mixed in 2 mL DMF. The reaction was stirred at room temperature for 2 h. Solvent was removed under vacuum. 14.0 mg of product XYIMSR-06 was obtained as a white powder after HPLC purification. Yield was 83%. HPLC conditions: column Phenomenex, Luna 10 × 250 mm, 10 μ. 25/75/0.1 MeCN/H_2_O/TFA, flow 10 mL/min. Product eluted at 12.0 min. ^1^H-NMR (500 MHz, DMSO-d_6_): δ 12.98 (s, 1H), 12.63 (br. 2H), 9.60 (m, 1H), 9.15 (br. s, 2H), 8.31 (s, 2H), 8.16 (d, J = 8.0, 1H), 8.05 (d, J =7.9, 1H), 7.90 (d, J = 8.1, 1H), 7.88 (t, J = 6.0, 1H), 7.83 (s, 1H), 7.69(br, 1H), 7.41 (d, J = 8.0 Hz, 2H), 7.19 (d, J = 8.0 Hz, 2H), 6.92 (d, J = 8.4, 4H), 6.64 (d, J =8.4, 4H), 6.50 (br, 2H), 4.52 – 4.46 (m, 2H), 4.24 (t, J = 7.2, 2H), 4.17 (td, J = 8.3, 5.5, 1H), 4.0-2.90 (overlaps with water signal), 2.65 (t, J = 7.5, 2H), 2.64 – 2.55 (m, 4H), 2.47 – 2.41 (m, 2H), 2.17 (t, J = 8.2, 2H), 1.94 (m, J = 7.5, 2H), 1.88 – 1.82 (m, 2H), 1.75 (m, J = 7.5, 2H), 1.66 – 1.60 (m, 1H), 1.53 – 1.46 (m, 1H), 1.45 (s, 3H), 1.28 – 1.17 (m, 2H). MS, calculated for C_65_H_89_N_16_O_21_S_3_^+^ [M+H]^+^: 1525.5545; Found: 1525.5527

2,2′,2″-(2-(4-(3-((11S,15S,19S)-15,19-Dicarboxy-24,24-bis(4-hydroxyphenyl)-10,13,17,21-tetraoxo-11-(4-(4-(4-oxo-4-((5-sulfamoyl-1,3,4-thiadiazol-2-yl)amino)butyl)-1H-1,2,3-triazol-1-yl)butyl)-3,6-dioxa-9,12,16,20-tetraazapentacosyl)thioureido)benzyl)-1,4,7-triazonane-1,4,7-triyl)triacetic acid([^63/65^Cu]XYIMSR-06). XYIMSR-06 1 mg (0.0007mmol) was dissolved in 0.5 mL 0.2 M NaOAc solution. To the solution 0.2 mg Cu(NO_3_)_2_3H_2_O was added in 0.1 mL of water. The reaction was heated at 100°C for 10 min and then loaded onto HPLC for purification. 0.5 mg of product was obtained as blue crystals. Yield was 48%. HPLC conditions: column Phenomenex, Luna 10 × 250 mm, 10 μ. 25/75/TFA MeCN/H_2_O/TFA, flow 10 mL/min. Product eluted at 8.3 min with the starting material eluting at 12.6 min. MS, calculated for C_65_H_87_CuN_16_O_21_S_3_^+^ [M+H]^+^: 1586.4684; Found: 1586.4683.

### Radiosynthesis

[^64^Cu]XYIMSR-06. 40 μg of XYIMSR-06 in 20 μL 0.2 M NaOAc solution was added to 60 μL ^64^CuCl_2_with 0.16 – 0.26 GBq (4.2 – 6.9 mCi) of radioactivity. The reaction was heated in a water bath at 65°C and pH 5.5−6 for 0.5 h. The reaction was then diluted with 1.5 mL of water and injected onto the HPLC for purification. Baseline separation was achieved between [^64^Cu]XYIMSR-06 and XYIMSR-06 with [^64^Cu]XYIMSR-06 eluting first. The average non-decay corrected radiochemical yield was 51.0 ± 4.5% (n=5) and specific activities were 4.1 – 8.9 GBq/μmol (110-240 Ci/mmol). HPLC conditions: column Phenomenex, Luna 10 × 250 mm, 10 μ. 23/77/TFA MeCN/H_2_O/TFA, flow 4 mL/min. Product eluted at 29.2 min. The collected radioactivity was diluted with 20 mL of water and loaded onto activated Sep-Pak (WAT020515, Waters, Milford MA). After the Sep-Pak was washed with 10 mL of water, [^64^Cu]XYIMSR-06 was eluted with 2 mL of ethanol. The ethanol was evaporated under a gentle stream of N_2_ (to a total volume of < 50 μL). The resulting solution was formulated in saline for the imaging and biodistribution studies.

### Cell lines and mouse models

Animal experiments were performed in accordance with protocols approved by the Johns Hopkins Animal Care and Use Committee (ACUC). Six-week-old female NOD/SCID mice were purchased from the Animal Resource Core of the Sidney Kimmel Comprehensive Cancer Center of Johns Hopkins and were subcutaneously injected in the upper right flank with 1 × 10^6^ SK-RC-52 cells in RPMI 1640 GlutaMAX™ media (Life Technologies, Frederick, MD) supplemented with 1% fetal bovine serum (FBS). Mice were monitored for tumor size and used for PET/CT imaging when the size of the tumor reached 100 mm^3^.

### Competitive fluorescence polarization assay [28, 45]

Fluorescence polarization (FP) experiments were performed in 21 μL of the assay buffer (12.5 mM Tris-HCl, pH 7.5, 75 mM NaCl) in transparent flat bottom 384 well Small Volume™ LoBase Microplates (Greiner Bio-One, Frickenhausen Germany) [29]. The FP reaction employed 100 nM of purified CAIX (R&D systems, Minneapolis, MN) and 80 nM FITC-labeled ligand within the assay buffer. The FP values were measured as mP units using the Safire2™ plate reader (Tecan, Morrisville, NC) with excitation at 475 nm and emission at 532 nm emission. 100 nM CAIX was incubated with serially diluted (from 8 μM to 488.2 fM) concentrations of the three targeting molecules, 1 and [^63/65^Cu]XYIMSR-06 for 30 min at room temperature in 384 well plates. 80 nM FITC-labeled ligand was added to each well and the reaction was incubated for 30 min at room temperature followed by FP measurements. Experiments were carried out in triplicate and the concentration resulting in 50% response (IC_50_) was calculated in GraphPad Prism 5 (GraphPad Software, La Jolla, CA) using the sigmoidal dose-response regression function.

### Biodistribution

Mice bearing SK-RC-52 xenografts within the upper right flank were injected intravenously with 740 kBq (20 μCi) of [^64^Cu]XYIMSR-06 in 200 μL of PBS. For *in vivo* competition (binding specificity) studies, tumor-bearing mice were injected with 740 kBq (20 μCi) of [^64^Cu]XYIMSR-06 and 200 nmole of 1 in 200 μL of PBS concurrently. At specific times after injection (1 h, 4 h, 8 h and 24 h), mice (n = 5) were sacrificed by cervical dislocation with blood immediately collected by cardiac puncture. Heart, lungs, pancreas, spleen, fat, brain, muscle, small intestines, liver, stomach, kidney, urinary bladder, and tumor were also collected. Each organ was weighed and the tissue radioactivity was measured with an automated gamma counter (1282 Compugamma CS, Pharmacia/ LKBNuclear, Inc., Mt. Waverly, Vic. Australia). The percentage of injected dose per gram of tissue (%ID/g) was calculated by comparison with samples of a standard dilution of the initial dose. All measurements were corrected for radioactive decay. Data are expressed as mean ± standard deviation (SD).

### Imaging

Mice harboring subcutaneous SK-RC-52 tumors within the upper right flank were injected intravenously (tail vein) with 22.2 MBq (600 μCi) of [^64^Cu]XYIMSR-06 in 250 μL of PBS (pH = 7.0). Anesthesia was then induced with 3% isoflurane and maintained with 2% isoflurane. Physiologic temperature was maintained with an external light source while the mouse was on the gantry. Whole body, 2-bed PET/CT imaging was performed using the SuperArgus small animal PET/CT scanner (Sedecal, Madrid, Spain), CT employing a 250-700 keV energy window. PET acquisition times were: 5 min/bed position (1 h post-injection of [^64^Cu]XYIMSR-06); 10 min/bed position (4 and 8 h) and 20 min/bed position (24 h). PET images were co-registered with the corresponding 360-slice CT images. Imaging datasets were reconstructed using the 3D-FORE/2D-OSEM iterative algorithm with 2 iterations and 16 subsets, using the manufacturer's software. Imaging data sets were reconstructed using the manufacturer's software. Display of images utilized the software package PMOD (v3.3, PMOD Technologies Ltd, Zurich, Switzerland).

## SUPPLEMENTARY MATERIALS FIGURES AND TABLES


