# Supplementary material for: [64Cu]XYIMSR-06: A dual-motif CAIX ligand for PET imaging of clear cell renal cell carcinoma
Source: Oncotarget. 2016 Jul 14;7(35):56471–9. doi: 10.18632/oncotarget.10602 (PMC5302928; doi:10.18632/oncotarget.10602)
Supplement: Supplementary file 1 [file oncotarget-07-56471-s001.pdf]

[^{64}Cu]XYIMSR-06: A dual-motif caix ligand for pet imaging of clear cell renal cell carcinoma

SUPPLEMENTARY DATA

General chemistry and radiochemistry methods

Solvents and chemicals obtained from commercial sources were of analytical grade or better and used without further purification. Fmoc-protected azidolysine, HBTU, and N-α-fmoc-L-aspartic acid α-tert-butyl ester were purchased from Chem Impex International, Inc. (Wooddale, IL). Na^{18}F in saline was purchased from PETNET(Hackensack, NJ). [^{64}Cu]$CuCl_2$ was purchased from the University of Wisconsin. p-SCN-Bn-NOTA, was purchased from Macrocyclics, Inc. (Dallas, TX). NOTA-NHS ester was purchased from CheMatech(Dijon, France). Copper (II) nitrate, triethylsilane (Et_3SiH), N,N-diisopropylethylamine (DIEA), triethylamine (TEA), piperidine, 4,4-bis(4-hydroxyphenyl)valeric acid, copper iodide (CuI), and tris[(1-benzyl-1H-1,2,3-triazol-4-yl) methyl]amine (TBTA) were purchased from Sigma-Aldrich (Saint Louis, MO). Pre-loaded O-bis-(aminoethyl) ethylene glycol on trityl resin was purchased from EMD Millipore (Billerica, MA). Flash chromatography was performed using MP SiliTech 32-63 D 60Å silica gel purchased from Bodman (Aston, PA). Recombinant human CAIX was purchased from R&D Systems (Minnepolis, MN). ^{1}H NMR spectra were recorded on a Bruker Ultrashield 500 MHz spectrometer. Chemical shifts (δ) were reported in ppm downfield by reference to proton resonances resulting from incomplete deuteration of the NMR solvent. ESI mass spectra were obtained on a Bruker Daltonics Esquire 3000 Plus spectrometer (Billerica, MA).

HPLC purification of non-labeled compounds were performed using a Phenomenex C18 Luna 10 × 250 mm^2 column on an Agilent 1260 infinity LC system (Santa Clara, CA). HPLC purification of radiolabeled ligands were performed on another Phenomenex C18 Luna 10 × 250 mm^2 and a Varian Prostar System (Palo Alto, CA), equipped with a Varian ProStar 325 UV-Vis variable wavelength detector and a Bioscan (Poway, CA) Flow-count in-line radioactivity detector, all controlled by Galaxie software. The specific radioactivity was calculated as the ratio of the radioactivity eluting at the retention time of product during the preparative HPLC purification to the mass corresponding to the area under the curve of the UV absorption. The purity of tested compounds as determined by analytical HPLC with absorbance at 254 nm was > 95%.

Synthesis and characterization

2,2’-(7-((14S,18S,22S)-18,22-dicarboxy-27,27-bis (4-hydroxyphenyl)-2,13,16,20,24-pentaoxo-14-(4-(4-(4-oxo-4-((5-sulfamoyl-1,3,4-thiadiazol-2-yl)amino)butyl)-1H-1,2,3-triazol-1-yl)butyl)-6,9-dioxa-3,12,15,19,23-pentaazaoctacosyl)-1,4,7-triazonane-1,4-diyl)diacetic acid (XYIMSR-04). 1-SI (1)14 mg (0.0126 mmol), NOTA-NHS 10 mg (0.0151 mmol) and triethylamine 50 µL were mixed in 2 mL DMF. The reaction was stirred at room temperature for 2 hours. All the solvent was removed under vacuum. 9.0 mg product XYIMSR-04 was obtained as white powder, after HPLC purification. Yield is 52%. HPLC condition: column Phenomenex, Luna 10 × 250 mm, 10 µ. Gradient 10/90/0.1 to 50/50/0.1 MeCN/H_2O/TFA, 0 – 10 min, flow 10 mL/min. Product was eluded at 6.9 min. ^{1}H-NMR (500 MHz, DMSO-d6): δ 12.99 (s, 1H), 12.25 (br. 2H), 9.15 (br. s, 2H), 8.31 (s, 2H), 8.24(m, 1H), 8.16 (d, J = 8.0, 1H), 8.05 (d, J =7.9, 1H), 7.90 (d, J = 8.1, 1H), 7.88 (t, J = 6.0, 1H), 7.83 (s, 1H), 6.92 (d, J = 8.4, 4H), 6.64 (d, J =8.4, 4H), 6.50 (br, 2H), 4.52 – 4.46 (m, 2H), 4.24 (t, J = 7.2, 2H), 4.17 (td, J = 8.3, 5.5, 1H), 4.0-2.84 (overlap with water signal), 2.65 (t, J = 7.5, 2H), 2.64 – 2.55 (m, 4H), 2.47 – 2.41 (m, 2H), 2.17 (t, J = 8.2, 2H), 1.94 (m, J = 7.5, 2H), 1.88 – 1.82 (m, 2H), 1.75 (m, J = 7.5, 2H), 1.66 – 1.60 (m, 1H), 1.53 – 1.46 (m, 1H), 1.45 (s, 3H), 1.28 – 1.17 (m, 2H). MS, calculated for $C_{57}H_{81}N_{15}NaO_{20}S_2^+$ [M+Na]+: 1382.5; Found: 1382.8.

[Al19F] 2,2’-(7-((14S,18S,22S)-18,22-dicarboxy-27,27-bis(4-hydroxyphenyl)-2,13,16,20,24-pentaoxo-14-(4-(4-(4-oxo-4-((5-sulfamoyl-1,3,4-thiadiazol-2-yl)

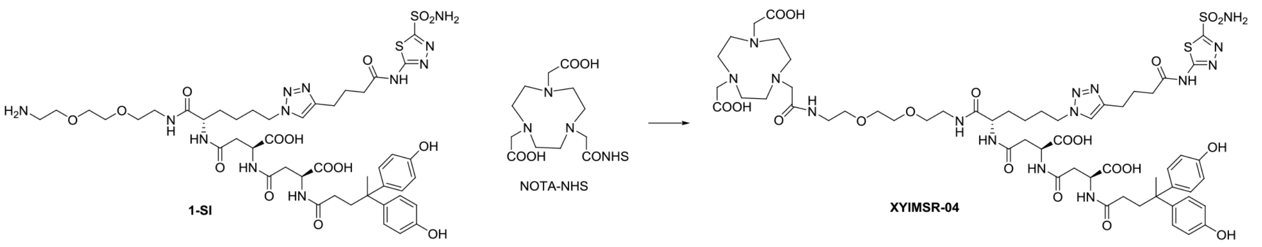

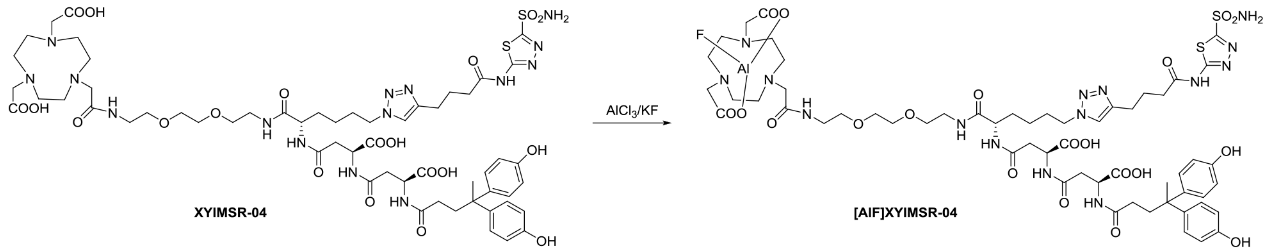

amino)butyl)-1H-1,2,3-triazol-1-yl)butyl)-6,9-dioxa-3,12,15,19,23-pentaazaoctacosyl)-1,4,7-triazonane-1,4-diyl)diacetic acid ([Al19F]XYIMSR-04). XYIMSR-04 1 mg (0.0007 mmol) was dissolved in 1 mL water/Ethanol 1:1. To the solution, 500 µL of $AlCl_3$ 2 mmol/NaOAc 2 mmol water solution (pH = 4) was added. Then, 500 µL of 10 mmol KF solution was added, together with 1 mL of ethanol. The resulting solution was heated at 110 °C for 30 min. 0.6 mg of [$Al^{19}F$]XYIMSR-04 was obtained as white crystal, after HPLC purification. Yield is 61 %. HPLC condition: column Phenomenex, Luna 10 x 250 mm, 10 µ. 20/80/TFA MeCN/H_2O/TFA, flow 4 mL/min. Product was eluded at 35.5 min. MS, calculated for $C_{57}H_{79}AlFN_{15}NaO_{20}S_2^+$ [M+Na]$^+$: 1426.4759; Found: 1426.4777.

Radiosynthesis

For convenience, $Na^{18}F$ in saline purchased from PETNET (Hackensack, NJ) was directly applied with to the synthesis with a slightly modified protocol (2). Due to the slow kidney clearance, we did not further optimize the reaction condition.

Radiosynthesis of [$Al^{18}F$]XYIMSR-04. 400µg of XYIMSR-04 in 200 µL water/Ethanol 1:1 was added to 1 mL of $Na^{18}F$ in 0.9% saline containing 4.6-14.0 mCi activity. To this mixture, 20 µL of 2 mM $AlCl_3$/NaOAc solution and 200 µL of ethanol were added. The reaction was kept at 105 °C for 20 min, then diluted to 2.0 mL with water and loaded onto a preparative HPLC column for purification. Retention times for the radiolabeled compound, [$Al^{18}F$]XYIMSR-04, and starting material, XYIMSR-04, were optimized to the point of baseline separation, with [$Al^{18}F$]XYIMSR-04 eluting first. [$Al^{18}F$]XYIMSR-04 was obtained a radiochemical yield of 4.3% (n=3) in specific radioactivity of 57-92 Ci/mmol in 1 hour. HPLC conditions: Phenomenex, Luna 10 x 250 mm, 10µ. 20/80/0.1 MeCN/H_2O/TFA, flow 4 mL/min. Product eluted at 35.5 min, while starting material eluted at 39.0 min.

Competitive fluorescence polarization assay

Binding of [$Al^{19}F$]XYIMSR-04 for CAIX was measured using a competitive fluorescence polarization (FP) assay as described in the experimental section. The IC_{50} values determined for dual motif precursor **1** (Figure 1, main text) and [$Al^{19}F$]XYIMSR-04 were 63.6 ± 2.8 and 96.7 ± 3.3 nM, respectively (Supplementary Figure S1).

Biodistribution

Mice bearing SK-RC-52 xenografts within the lower left flank were injected intravenously with 740 kBq (20 µCi) of [$Al^{18}F$]XYIMSR-04 in 200 µL of PBS. At specific 1 hour time point mentioned in the paper, mice (n = 5) were sacrificed by cervical dislocation and the blood was immediately collected by cardiac puncture. Heart, lungs, pancreas, spleen, fat, brain, muscle, small intestines, liver, bone, stomach, kidney, urinary bladder, and tumor were collected. Each organ was weighed and the tissue radioactivity was measured with an automated gamma counter (1282 Compugamma CS, Pharmacia/LKBNuclear, Inc., Mt. Waverly, Vic. Australia). The percentage of injected dose per gram of tissue (% ID/g) was calculated by comparison with samples of a standard dilution of the initial dose. All measurements were corrected for radioactive decay.

Data were expressed as mean ± standard deviation (SD). Prism software (GraphPAD, San Diego, California) was used to determine statistical significance. Statistical significance was calculated using a paired t test. *P*-values < 0.0001 were considered significant.

The biodistribution data at 1 h is shown below. The tumor uptake is 14.40%ID/g, with tumor-to –blood, -muscle and –kidney of 22.1, 9.74 and 0.28.

Imaging

Mice harboring subcutaneous SK-RC-52 tumors with the lower left flank were injected with 7.4 MBq (200 µCi) of [$Al^{18}F$]XYIMSR-04 in 250 µL of PBS (pH = 7.0) intravenously (tail vein). Anesthesia was then induced with 3% isofluorane and maintained at 2% isoflurane. Physiologic temperature was maintained with an external light source while the mouse was on the gantry. Imaging employed a CT-equipped Gamma Medica-Ideas SPECT scanner (Northridge, CA). Whole body 2-bed PET scan was performed using ARGUS small-animal PET/CT scanner (Sedecal, Madrid, Spain) at 250-700 keV energy window.

PET acquisition times were: 5 min/bed (1 h) post-injection of [Al^{18}F]XYIMSR-04. PET images were co-registered with the corresponding 360-slice CT images. Imaging datasets were reconstructed using the 3D-FORE/2D-OSEM iterative algorithm with 2 iterations and 16 subsets, using the manufacturer's software. Display of images utilized Amide software (Dice Holdings, Inc. NY). The 1 h image is shown below (Supplementary Figure S2):

Metabolism study

In vivo metabolism of [^{64}Cu]XYIMSR-06 in NSG mice intravenously (tail vein) with 7.4 MBq (200 µCi) of [^{64}Cu]XYIMSR-06 in 200 µL of PBS (pH = 7.0). One mouse was euthanized at 0.5, 1, and 2hr time points and plasma was prepared by centrifugation of whole blood for 15 minutes at 2,500xg at 4°C. 5 uL of the plasma samples and control (7.4 kBq of [^{64}Cu]XYIMSR-06) were spotted onto silica gel 60 RP-18 F254S glass TLC plates (EMD Millipore Corp., Billerica, MA) and the plates were developed using a mobile phase consisting of 45% acetonitrile, 55% water and 0.1% trifluoroacetic acid. The TLC plate was dried and exposed to Kodak Biomax x-ray film (Fisher Scientific). Standard solutions had Rf value of 0.6. The intensity of bans on the film were calculated with the Image J software (NIH, Bethesda, MD). The percentages of intact [^{64}Cu]XYIMSR-06 in plasma were 86.2%, 98.3, and 100% for 0.5h, 1h, and 2h plasma samples, respectively.

REFERENCES

1. Wichert M, Krall N, Decurtins W, Franzini RM, Pretto F, Schneider P, Neri D, Scheuermann J. Dual-display of small molecules enables the discovery of ligand pairs and facilitates affinity maturation. Nature chemistry. 2015;7:241-9.
2. D'Souza CA, McBride WJ, Sharkey RM, Todaro LJ, Goldenberg DM. High-yielding aqueous 18F-labeling of peptides via Al18F chelation. Bioconjugate chemistry. 2011;22:1793-803.

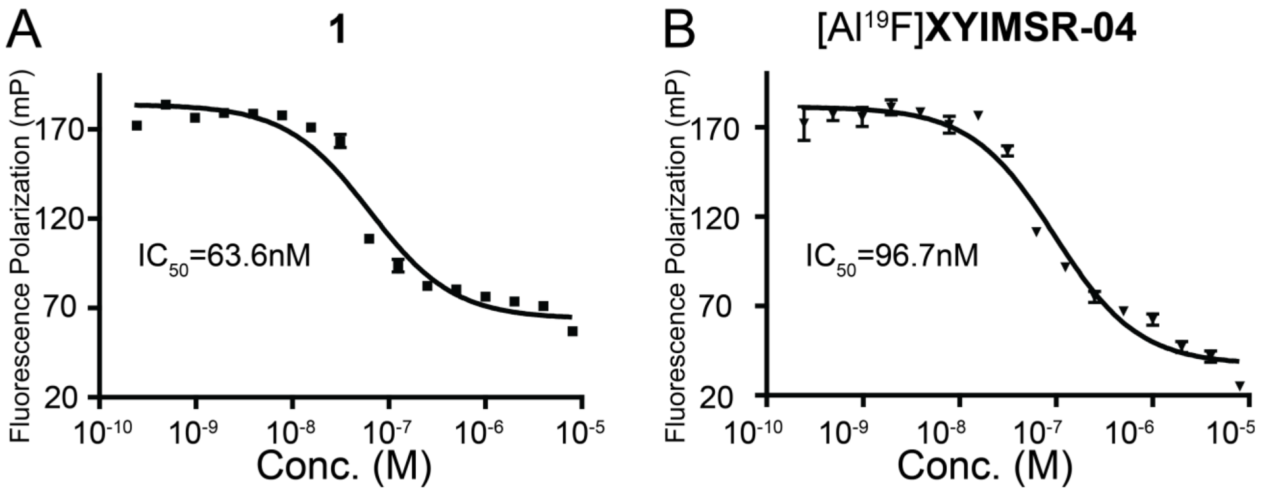

Supplementary Figure S1: [Al^{19}F]XYIMSR-04 demonstrates high binding affinity to CAIX. IC_{50} values of positive control **1** and [Al^{19}F]**XYIMSR-04** was determined via measuring inhibition of fluorescent polarization of FITC labeled compound.

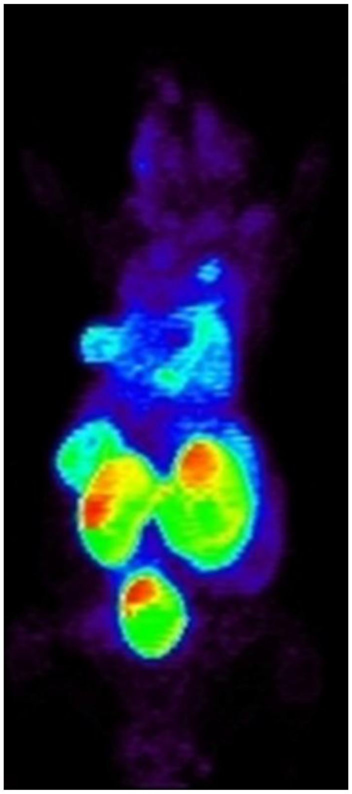

Supplementary Figure S2: PET imaging of [$Al^{18}F$]XYIMSR-04 at 1 h post-injection.

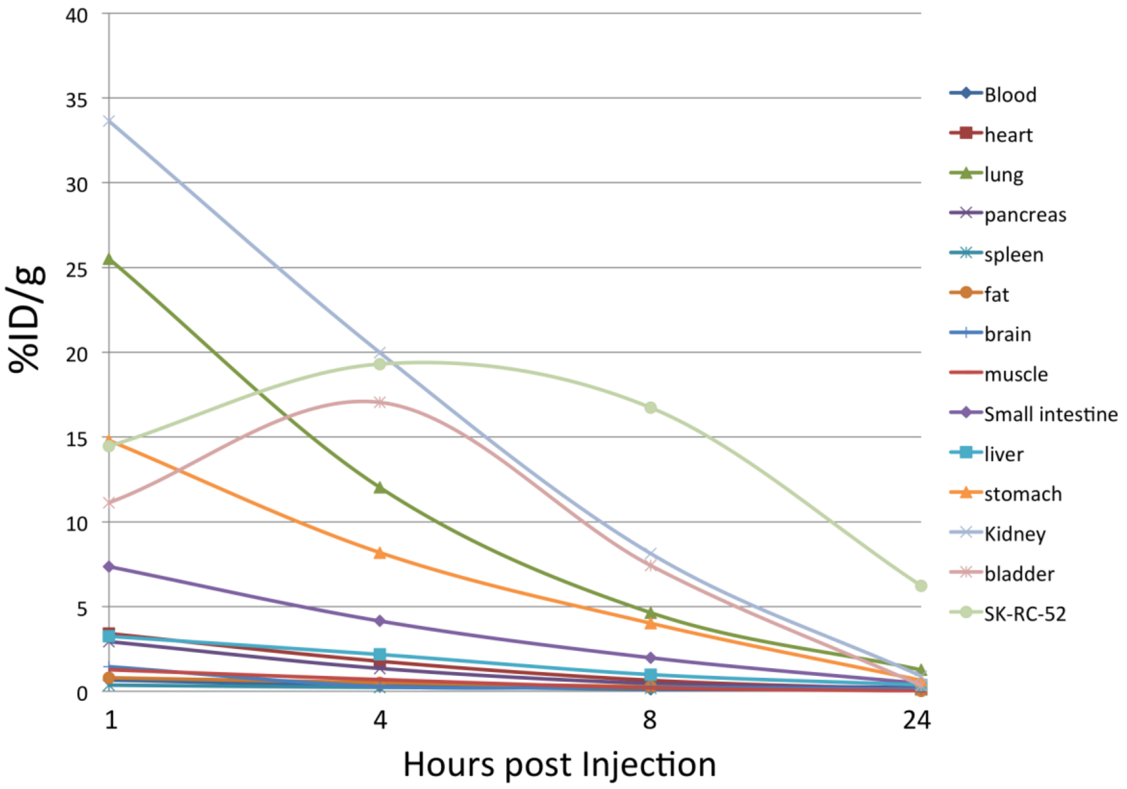

Supplementary Figure S3: Time-activity curve of [^{64}Cu]XYIMSR-06 for major organs and SK-RC-52 tumor.

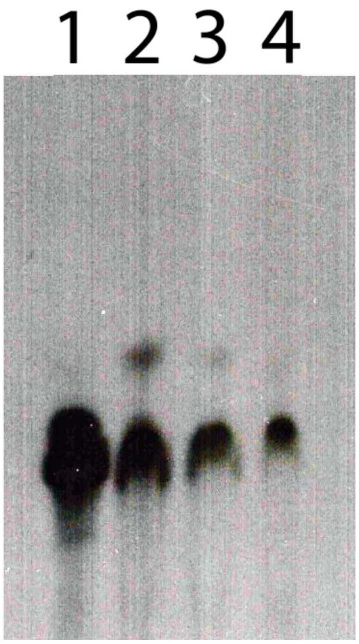

Supplementary Figure S4: TLC analysis of metabolism of [^{64}Cu]XYIMSR-06 in mice. 1: control, 2: 0.5h plasma, 3: 1h plasma, 4: 2h plasma.

Supplementary Table S1: Biodistribution of [Al^{18}F]XYIMSR-04

Organs	1 h (%ID/g)
Blood	0.65 ± 0.11
Heart	5.03 ± 0.51
Lung	34.07 ± 5.85
Pancreas	3.47 ± 0.16
Spleen	0.54 ± 0.18
Fat	1.54 ± 0.25
Brain	0.66 ± 0.04
Muscle (mm)	1.74 ± 0.58
Sm. intestine	9.59 ± 1.04
Liver	5.55 ± 0.91
Stomach	21.62 ± 1.85
Kidney (kid)	50.84 ± 2.65
Bladder	20.12 ± 12.51
Bone	1.91 ± 1.39
Tumor	14.40 ± 2.18
Tumor/Blood	22.1 ± 1.5
Tumor/mm	9.7 ± 2.9
Tumor/kid	0.28 ± 0.03

Supplementary Table S2: Calculated half-life of [^{64}Cu]XYIMSR-06 for major organs and SKRC-52 tumor

	Half-life (hr)	Organ	Half-life (hr)
Blood	2.153	Muscle	3.035
Heart	2.293	Small intestine	3.322
Lung	2.542	Liver	3.375
Pancreas	2.330	Stomach	3.397
Spleen	4.008	Kidney	3.591
Fat	4.666	Bladder	116.2
Brain	0.860	SK-RC-52	996.1

Half-life for each organ was calculated in GraphPad Prism 4 (GraphPad Software, La Jolla, CA) using the sigmoidal dose-response one phase exponential decay function.
